# Associations of Metabolic Genes (*GSTT1*, *GSTP1*, *GSTM1*) and Blood Mercury Concentrations Differ in Jamaican Children with and without Autism Spectrum Disorder

**DOI:** 10.3390/ijerph18041377

**Published:** 2021-02-03

**Authors:** Mohammad H. Rahbar, Maureen Samms-Vaughan, Sepideh Saroukhani, Jan Bressler, Manouchehr Hessabi, Megan L. Grove, Sydonnie Shakspeare-Pellington, Katherine A. Loveland, Compton Beecher, Wayne McLaughlin

**Affiliations:** 1 Department of Epidemiology, Human Genetics, and Environmental Sciences (EHGES), School of Public Health, The University of Texas Health Science Center at Houston, Houston, TX 77030, USA; Sepideh.Saroukhani@uth.tmc.edu (S.S.); Jan.Bressler@uth.tmc.edu (J.B.); Megan.L.Grove@uth.tmc.edu (M.L.G.); 2 Biostatistics/Epidemiology/Research Design (BERD) Component, Center for Clinical and Translational Sciences (CCTS), The University of Texas Health Science Center at Houston, Houston, TX 77030, USA; Manouchehr.Hessabi@uth.tmc.edu; 3 Division of Clinical and Translational Sciences, Department of Internal Medicine, McGovern Medical School, The University of Texas Health Science Center at Houston, Houston, TX 77030, USA; 4 Department of Child & Adolescent Health, Mona Campus, The University of the West Indies (UWI), Kingston 7, Jamaica; msammsvaughan@gmail.com (M.S.-V.); sydonniesp@gmail.com (S.S.-P.); 5 Human Genetics Center, School of Public Health, The University of Texas Health Science Center at Houston, Houston, TX 77030, USA; 6 Louis A Faillace, MD, Department of Psychiatry and Behavioral Sciences, McGovern Medical School, The University of Texas Health Science Center at Houston, Houston, TX 77054, USA; Katherine.A.Loveland@uth.tmc.edu; 7 Department of Basic Medical Sciences, Mona Campus, The University of the West Indies, Kingston 7, Jamaica; compton.beecher@uwimona.edu.jm (C.B.); wayne.mclaughlin@uwimona.edu.jm (W.M.); 8 Caribbean Genetics (CARIGEN), Mona Campus, The University of the West Indies, Kingston 7, Jamaica

**Keywords:** autism spectrum disorder (ASD), blood mercury concentrations, glutathione S-transferase (GST) genes, seafood consumption, interaction, Jamaica

## Abstract

We investigated interactive roles of three metabolic glutathione S-transferase (GST) genes (*GSTP1*, *GSTT1*, and *GSTM1*) and autism spectrum disorder (ASD) status in relation to blood Hg concentrations (BHC) of Jamaican children. We used data from 266 children (2-8 years) with ASD and their 1:1 age- and sex-matched typically developing (TD) controls. After adjusting General Linear Models for child’s age, socioeconomic status, consumption of leafy vegetables, fried plantain, canned fish, and the interaction between *GSTP1* and *GSTT1*, we found significant interactions between *GSTP1* and ASD status in relation to BHC either in a co-dominant or dominant genetic model for *GSTP1*(*P* < 0.001, *P* = 0.007, respectively). In the co-dominant model for the Ile105Val *GSTP1* polymorphism, geometric mean (GM) BHC in ASD cases with genotype Ile/Ile were significantly higher than in cases with the Ile/Val genotype (0.73 vs. 0.48 µg/L, *P* = 0.01). In contrast, in TD controls with the Ile/Val genotype GM BHC were significantly higher than in those with the Ile/Ile genotype (0.72 vs. 0.49 µg/L, *P* = 0.03) or the Val/Val genotype (0.72 vs. 0.51 µg/L, *P* = 0.04). Although our findings are consistent with the role of *GSTP1* in detoxification of Hg, replication in other populations is warranted.

## 1. Introduction

Mercury (Hg) is a toxic metal that exists in elemental, inorganic and organic chemical forms and has harmful effects on human health [1,2]. The organic form of Hg (methylmercury) is the most toxic form of Hg in humans and exposure to methylmercury primarily occurs through the diet, particularly consumption of fish and fish products [3]. Other common routes of elemental and inorganic Hg exposures are dental amalgam [4,5], dermal contact, exposure through air pollution from Hg released in the air from fossil fuel combustion, mining, and smelting [1], industrial waste, as well as occupational and ritualistic practices [3]. As a neurotoxic agent, several studies have shown associations between high levels of Hg in various human tissues and central nervous system and neurodevelopmental disorders including impairments in vision and hearing, cerebral palsy, intellectual and neurodevelopmental disabilities, hyperactive tendon reflexes and general paralysis [6,7,8,9]. Other studies have reported significant associations between even lower levels of Hg and attention deficits and anxiety, as well as impairments in language, learning, and fine motor and visual–spatial organizational skills [8,10,11,12].

The possible association between exposure to Hg and autism spectrum disorder (ASD) has been investigated in several studies and conflicting findings have been reported. For example, some studies have found that individuals with ASD had significantly higher Hg levels versus typically developing (TD) controls as measured by levels in hair [13,14,15,16], urine [17], red blood cells [18,19,20], whole blood [21,22,23,24], and baby teeth [25]. In contrast, other studies reported significantly lower Hg levels in ASD cases than TD controls as measured in hair [26,27,28,29], or no significant difference in the Hg level between ASD cases and TD controls as measured in whole blood [30,31,32,33,34,35,36,37], hair [31,34], and urine [33,34,38,39,40]. In addition, findings of a meta-analysis that included 44 case-control studies indicated that while the Hg concentrations in whole blood (mean difference =0.43, 95% CI: 0.12, 0.74, *P* = 0.007), and red blood cells (mean difference =1.61, 95% CI: 0.83, 2.38, *P* < 0.001) were significantly higher in individuals with ASD compared to TD controls, the Hg level in hair (mean difference = 0.14 mg/g, 95% CI: −0.28, −0.01, *P* = 0.039) was significantly lower in ASD cases than TD controls [41]. Moreover, Hg concentrations in urine were not significantly different between children with and without ASD (0.51 mg/g creatinine, 95% CI: −0.14, 1.16, *P* = 0.121) [41]. Considering that all aforementioned findings are based on case-control studies, at least part of the reason for the inconsistencies could be related to potential confounding effects of dietary practices including fish consumption, as well as other sources of Hg exposure and sociodemographic characteristics on the association between Hg levels in different tissues and ASD. To our knowledge, among all the aforementioned studies, only a few had available information about frequencies of food consumption including fish and seafood [26,28,30,32,36]. However, only three studies have controlled for the confounding effect of food consumption and other sociodemographic characteristics while assessing the differences in Hg levels between ASD cases and TD controls [30,32,36]. All three of these studies have consistently reported no significant difference in the blood Hg concentrations between ASD cases and TD controls after controlling for fish or seafood consumption and other sociodemographic characteristics. Specifically, in the Childhood Autism Risk from Genetics and Environment (CHARGE) study, the geometric mean blood Hg concentrations in 2–5-year-old ASD cases were significantly lower than TD controls after adjusting for the child’s age and sex as well as maternal education and birth place (*P* = 0.02). However, after further adjustment for children’s fish consumption and other sources of Hg exposure, no significant difference (*P* = 0.75) in the geometric mean blood Hg concentrations between ASD cases (0.19 μg/L) and TD controls (0.28 μg/L) was observed [30]. Another study used data from the CHARGE study to develop a toxicokinetic model that incorporates both blood Hg concentrations at birth, as well as self-reported data on fish consumption before and throughout pregnancy to estimate the cumulative exposure to MeHg [36]. They have reported no association between cumulative MeHg exposure and ASD after adjusting for sociodemographic status as a potential confounder (Odds Ratio (OR) = 0.95, 95% confidence interval (CI): 0.95, 1.12). Similarly, in our Jamaican Autism Study, even though based on univariable analysis of data from 65 pairs of sex- and age-matched ASD cases and TD controls, we observed that ASD cases had a significantly lower geometric mean blood Hg concentration than the TD control group (*P* = 0.02). However, after adjusting for potential confounding factors including the child’s frequency of seafood consumption, maternal age, and parental education, the association between Hg and ASD was no longer statistically significant (*P* = 0.61) [32].

In addition to the role of diet in blood Hg concentrations, several studies have suggested variation in detoxification and excretory mechanisms as an explanation for differences in Hg concentrations in children with and without ASD [13,18,28,29,41,42,43]. Possible neurotoxic mechanisms of Hg include the induction of chronic oxidative stress and impairment of mitochondrial functioning [44,45,46]. Glutathione S-transferases (GST) are a family of phase II enzymes that are the main endogenous protectors of cells from oxidative stress by their critical role in detoxification of exogenous chemicals (e.g., heavy metals) [47], and endogenous metabolites that are associated with oxidative stress [48,49,50].

Metabolic GST genes, including *GSTP1*, *GSTM1*, and *GSTT1* encode GST enzymes and are highly polymorphic [50]. It has been demonstrated that null alleles of some GST genes including *GSTM1* and *GSTT1* can interrupt the function of these enzyme and consequently decrease the detoxification capacity [51]. For example, there is a growing evidence from epidemiological and experimental studies suggesting that genetic polymorphisms in GST genes can heavily influence the susceptibility to the cytotoxic consequences of Hg [52,53,54,55]. These data are particularly interesting because the presence of chronic oxidative stress, genetically determined defects of the glutathione system, and mitochondrial dysfunction in the blood and brain have also been reported in children with ASD [56,57,58,59,60,61,62]. All of this evidence indicates that genetic variation conveys a possible differential susceptibility to environmental exposure to Hg in relation to neurodevelopment.

Since 2009, in collaboration with faculty at the University of the West Indies (UWI), Mona campus, in Jamaica, our research team at the University of Texas Health Science Center at Houston (UTHealth) has investigated the additive and interactive associations of six heavy metals including Hg and GST genes (*GSTP1*, *GSTT1*, and *GSTM1*) in relation to ASD in Jamaican children. As mentioned earlier, we have previously reported that blood Hg concentrations were not associated with ASD in a multivariable model that adjusted for the child’s frequency of seafood consumption, parental education, and maternal age as potential confounding factors [32]. However, we have recently reported a marginally significant interaction between *GSTP1* and a mixture of three metals (Pb, Hg, and Mn) in relation to ASD (*P* = 0.07) [63], though when analyzed as an individual metal (i.e., not in a mixture), the interaction between *GSTP1* and blood Hg concentrations in relation to ASD was not significant, (*P* = 0.12) [63]. Under the co-dominant genetic model, we have also found that among children who were heterozygous for the *GSTP1* Ile105Val polymorphism, the odds of ASD for those with the *GSTT1* DD genotype was three times that of those with either the *GSTT1* I/I or I/D genotype (*P* = 0.03), suggesting interaction between *GSTP1* and *GSTT1* in relation to ASD [64]. In addition, we have previously reported that the associations between *GSTP1* genotype and blood arsenic concentrations [65], as well as blood aluminum concentrations [66] significantly vary by ASD status (*P* = 0.04 and *P* < 0.02 for the interaction between *GSTP1* and ASD status in relation to blood arsenic and aluminum concentrations, respectively). Considering the evidence from the literature and our previous findings about the complexity of the role of diet and metabolic genes in blood concentrations of heavy metals in children with and without ASD, we propose that metabolic GST genes may have a role in susceptibility to environmental exposure to Hg. In this study, we investigate the possible interaction of each of the three metabolic GST genes (*GSTP1*, *GSTM1*, and *GSTT1*) with ASD status, and possible pair-wise gene–gene interactions of these genes in relation to blood Hg concentrations of Jamaican children.

## 2. Materials and Methods

### 2.1. General Description

The Epidemiological Research on Autism in Jamaica (ERAJ) and ERAJ-Phase2 (ERAJ-2) age- and sex-matched case-control studies began enrollment of children 2–8 years old with ASD and their age- and sex-matched TD controls in December 2009, to investigate the potential individual or interactive associations of environmental exposures and three GST genes (*GSTP1, GSTT1,* and *GSTM1*) in relation to ASD in Jamaican children. The process for recruitment and assessment of ASD cases and TD controls has been reported earlier [67,68,69,70]. In brief, after obtaining written consent from parents and child assent when applicable, we administered the Autism Diagnostic Observation Schedule-Second Edition (ADOS-2) [71] and Autism Diagnostic Interview-Revised (ADI-R) [72] to ascertain ASD status in suspected ASD cases recruited from the Jamaican Autism Database, and the Social Communication Questionnaire (SCQ) [73] to rule out developmental disorders (SCQ score ≤ 6) in the TD controls. In addition, at the time of enrollment we administered a socioeconomic status (SES) questionnaire to assess socio-demographic characteristics including parents’ educational levels and the family’s SES that is measured by ownership of a car in Jamaica. We also administered a food frequency questionnaire to assess potential dietary sources of exposure to heavy metals through the types and frequency of seafood, vegetables and fruits consumed by the children on a weekly basis. We classified the types of seafood, vegetables, and fruits based on their characteristics and species. For example, we classified different types of vegetables as follows: (1) leafy vegetables in three classes: (lettuce), (callaloo, broccoli, or pak choi), and (cabbage); (2) root vegetables in two classes: (yam, sweet potato, or dasheen) and (carrot or pumpkin); and (3) legumes. We considered the frequency of different types of food consumed as a categorical variable (consumed and never consumed) for analysis. Additional details regarding the food categories have been reported previously [67].

To assess exposure to the heavy metals including Hg and to determine GST gene genotypes, we collected 4-5 mL of whole blood from each child at the end of the interview and other assessments. In the current study, we used data from 266 Jamaican children with ASD, and their 1:1 age (±6 months) and sex-matched TD controls [n = 532 that includes 266 matched pairs] who were enrolled in the ERAJ studies between December 2009 and September 2017. The study protocol was conducted in accordance with the Declaration of Helsinki and approved by the Institutional Review Boards (IRBs) of UTHealth (HSC-SPH-09-0059), UWI, and Michigan Department of Health and Human Services (MDHHS).”

### 2.2. Assessment of Mercury Exposures

The Trace Metals Lab, a Centers for Disease Control and Prevention (CDC) certified lab at the Michigan Department of Health and Human Services (MDHHS) in Lansing, Michigan, USA, conducted the blood Hg concentration analyses. The Caribbean Genetics (CARIGEN) lab at UWI processed whole venous blood samples, each of which was about 2–3 mL, and shipped the samples to the Trace Metals Lab at MDHHS for assay of several heavy metals including Hg. Since the technology to detect metal concentrations in blood samples has changed over the last 11 years, the MDHHS reported different limits of detection (LoD) for Hg in phases 1 & 2 of the ERAJ study (i.e., LoD for Hg was 0.3 μg/L in phase 1 and 0.25 μg/L in phase 2). In this study, MDHHS reported 16.7% of blood Hg concentrations as undetectable because they were below the LoD. According to an established Quality Control (QC) program at MDHHS Trace Metal Lab, bovine blood spiked with known quantities of mercury were included as controls for mercury levels in the blood samples. All samples were diluted and analyzed on a PerkinElmer Elan DRC II inductively coupled plasma mass spectrometer (PerkinElmer, Waltham, MA, USA).

### 2.3. Genetic Analysis

In brief, regions of the *GSTM1* and *GSTT1* genes were amplified in two independent TaqMan Copy Number Assay reactions, *GSTM1* Assay ID: Hs02575461_cn and *GSTT1* Assay ID: Hs00010004_cn (www.thermofisher.com), to detect insertion/deletion polymorphisms. *GSTM1* and *GSTT1* homozygous deletions were coded as DD and the presence of an insertion was coded as I*. Assessment of the *GSTP1* Ile105Val polymorphism (rs1695) was carried out using the TaqMan Drug Metabolism SNP genotyping assay C_3217198_20. All three assays have been described in detail previously [64,70,74].

### 2.4. Statistical Analysis

We compared demographic and SES characteristics of ASD cases and TD controls using descriptive analyses. For the purpose of analysis all Hg concentrations below LoD were imputed by calculating LoD/($\sqrt{2}$) [75]. Since the distribution of blood Hg concentrations was skewed, we used the natural logarithm (ln) to transform data and produce an approximately normal distribution. We applied the natural exponential function to the means of the log transformed blood Hg concentrations to transform them back to their original scale (i.e., µg/L) and are referred to as the geometric means.

There are three genotypes (Ile/Val, Ile/Ile, Val/Val) for the *GSTP1* Ile105Val polymorphism. The *GSTP1* gene was analyzed assuming three different genetic models: co-dominant (Ile/Ile, Ile/Val, and Val/Val), dominant (Ile/Ile vs Val/*), and recessive (Ile/* vs. Val/Val). We also tested whether the *GSTP1* polymorphism met Hardy-Weinberg equilibrium expectations using the Chi-square test in the TD control group. Since the genotyping assay does not distinguish between a normal homozygote (I/I) and a heterozygote (I/D) for *GSTT1* and *GSTM1*, we considered only a recessive model using a binary variable: I/* (I/I or I/D) and DD (null allele).

In this study, using conditional logistic regression (CLR) models, we compared the distributions of socio-demographic characteristics, and consumption of various types of food between ASD case and TD control groups. We also used CLRs to assess possible associations between various exposure variables including GST genotypes (*GSTP1*, *GSTT1*, and *GSTM1*) and ASD status.

Univariable General Linear Models (GLMs) with the log-transformed blood Hg concentrations as the dependent variable were used to investigate the possible role of the three GST genes, ASD status, sociodemographic characteristics, and consumption of various types of food in determining these levels. In all GLMs, we included an appropriate number of dummy variables representing the matched pairs to control for the clustering effect of matching (e.g., 265 dummy variables for 266 matched pairs). To investigate the relationship between the genotypes for each of the three GST genes and blood Hg concentrations within the ASD case and TD control groups, we used multivariable GLMs and included the interactions between each of the three GST genes and ASD status in the model. In addition, in adjusted multivariable GLMs, we included child’s age [76], as well as SES, consumption of leafy vegetables (callaloo, broccoli, or pak choi), fried plantain, and canned fish (sardine or mackerel fish) as important covariates that have been identified to be associated with ASD status and blood Hg concentrations previously [32]. Furthermore, using multivariable GLMs, we assessed two-way interactions between genotypes of the three GST genes in relation to blood Hg concentrations. Since we found a significant interaction between the *GSTP1* and *GSTT1* genes in relation to blood Hg concentrations, in addition to the aforementioned covariates, we accounted for this gene-gene interaction in the adjusted multivariable GLMs that involved the *GSTP1* or *GSTT1* genes. Using the CONTRAST statement in PROC GLM in SAS [77] we tested whether the geometric mean blood Hg concentrations were significantly different between individuals with various GST genotypes, separately for ASD cases and TD controls. Similarly, we tested the statistical significance of the difference in the geometric mean blood Hg concentrations between ASD cases and TD controls by GST genotype. Unadjusted and adjusted geometric mean of the blood Hg concentrations were calculated for both groups of children (ASD and TD) with different GST genotypes. All statistical tests were performed at 5% level of significance without making any adjustments for multiple comparisons. We conducted all analyses using SAS software [78].

## 3. Results

At the time of enrollment, the age of 30.5% of ASD cases and 28.6% of the TD controls was 72 months or older. Nearly all of the ASD cases (95.5%) and TD controls (97.0%), and their parents (>96%) were Afro-Caribbean. At the time of the child’s birth, a significantly higher proportion of the mothers of ASD cases were age 35 years or older compared to mothers of TD controls (18.9% vs. 12.3%). Similarly, a higher proportion of ASD cases (62%) had at least one parent with education beyond high school compared to the TD controls (48%). ASD cases were more likely to be from families with higher SES compared to TD controls (56.4% versus 39.9% car ownership by ASD cases and TD control families, respectively). For TD children in our data, the frequency of the *GSTM1* and *GSTT1* null genotypes among TD controls were 23.6% and 24.8%, respectively. Furthermore, the frequencies of the *GSTP1* genotypes in the TD children were in agreement with Hardy-Weinberg equilibrium expectations (*P* = 0.28). In addition, the frequencies of *GSTM1*, *GSTP1*, and *GSTT1* genotypes were not significantly different between ASD cases and TD controls (all *P* > 0.11). The arithmetic mean blood Hg concentration was 1 µg/L for both children with ASD and TD children, indicating no significant difference between these two groups, (*P* = 0.93) (Table 1).

In a comparison of food consumption between ASD cases and TD controls, we observed that a significantly lower proportion of ASD cases reported eating root vegetables (carrot or pumpkin) [Matched Odds Ratio (MOR) = 0.52, 95% CI: (0.33, 0.82), *P* = 0.01], and other types of fruits and vegetables (all *P* ≤ 0.01). In addition, compared to TD controls, ASD cases consumed significantly less servings of canned fish (sardine and mackerel) [MOR (95% CI) = 0.53 (0.34, 0.82), *P* < 0.01], salted fish [MOR (95% CI) = 0.49 (0.33, 0.72), *P* < 0.01], and shellfish [MOR (95% CI) = 0.35 (0.18, 0.68), *P* < 0.01]. The frequency distributions of eating other types of food between ASD and TD children are shown in Table 2.

The univariable GLM that compared geometric mean blood Hg concentrations between children with various levels and types of exposures revealed a significant association between blood Hg concentrations and ASD status (geometric mean blood Hg concentration for ASD group = 0.62 µg/L vs. 0.76 µg/L for the TD control group, *P* < 0.01) (Table 3). Blood Hg concentrations were also significantly associated with child’s age at enrollment (*P* < 0.05), and consumption of root vegetables [yam, sweet potato, or dasheen (*P* = 0.03)], leafy vegetables [callalloo, broccoli, or pak choi (*P* = 0.02); cabbage (*P* = 0.03)], as well as some types of fruits [tomatoes (*P* < 0.01); fried plantains (*P* < 0.01)]. Similarly, we found significant associations with high seafood consumption (more than 6 meals per week), consumption of salt water fish, canned fish (sardine and mackerel), and salted fish (all *P* < 0.05). Blood Hg concentrations were not significantly associated with *GSTT1* (*P* = 0.61), *GSTM1* (*P* = 0.71), or *GSTP1* polymorphisms (*P* > 0.14 for all pairwise comparisons) (Table 3).

In an unadjusted multivariable model that assessed the association between the *GSTP1* gene and blood Hg concentrations within the ASD case and TD control groups, we identified a significant interaction between *GSTP1* genotype and ASD status in relation to blood Hg concentrations using either a co-dominant (overall interaction *P* = 0.002) or dominant (*P* = 0.021) genetic model (Table 4). Specifically, in the co-dominant genetic model for *GSTP1*, the geometric mean blood Hg concentrations were significantly higher in ASD cases with genotype Ile/Ile than in those with genotype Ile/Val (0.87 µg/L vs. 0.54 µg/L, *P* < 0.01). We also found that the geometric mean blood Hg concentration in TD controls with genotype Ile/Val was significantly higher than in those with genotype Val/Val (0.89 µg/L vs. 0.57 µg/L, *P* < 0.01). Additionally, in the dominant model for *GSTP1*, ASD cases with the Ile/Ile genotype had a significantly higher geometric mean blood Hg concentration compared to those with the Ile/Val or Val/Val genotype (0.85 µg/L vs. 0.56 µg/L, *P* = 0.01). However, there were no significant associations between the *GSTP1* gene and blood Hg concentrations in the TD control group, when we used the dominant model for *GSTP1*. Specifically, TD control children with Ile/Val or Val/Val genotypes had a geometric mean blood Hg concentration of 0.78 µg/L compared to 0.70 µg/L for TD control children with the Ile/Ile genotype (*P* = 0.53). In the recessive model for *GSTP1*, although the interaction between the *GSTP1* gene and ASD status was not statistically significant (*P* = 0.11), we found that TD control children with genotype Val/Val had significantly lower geometric mean blood Hg concentrations than those with Ile/Ile or Ile/Val genotypes (0.57 µg/L vs. 0.83 µg/L, *P* = 0.03).

Similar findings were observed for the ASD case and TD control groups in the *GSTP1* co-dominant genetic model after accounting for the interaction between *GSTT1* and *GSTP1* and further adjustment for child’s age, SES, consumption of leafy vegetables (callaloo, broccoli, or pak choi), fried plantain, and canned fish (sardine or mackerel fish) as covariates (overall interaction *P* < 0.001 in the adjusted model). Specifically, using the co-dominant genetic model for *GSTP1*, while the adjusted geometric mean blood Hg concentration in ASD cases with genotype Ile/Ile was significantly higher than in those with genotype Ile/Val (0.73 µg/L vs. 0.48 µg/L, *P* = 0.01), TD control children with genotype Ile/Ile had a significantly lower adjusted geometric mean blood Hg concentration than those with genotype Ile/Val (0.49 µg/L vs. 0.72 µg/L, *P* = 0.03). We also found that TD control children with genotype Ile/Val had a significantly higher adjusted geometric mean blood Hg concentration than those with genotype Val/Val (0.72 µg/L vs. 0.51 µg/L, *P* = 0.04). Similarly, in the dominant model for *GSTP1*, the interaction between ASD status and the *GSTP1* gene in relation to blood Hg concentrations remained significant after adjusting for interaction between the *GSTT1* and *GSTP1* genes, and other aforementioned covariates (overall interaction *P* = 0.007 in the adjusted model). However, the difference between adjusted geometric mean blood Hg concentrations of ASD cases with the Ile/Ile genotype and those with the Ile/Val or Val/Val genotype became marginally significant (0.74 µg/L vs. 0.54 µg/L, *P* = 0.06). The interaction between ASD status and the *GSTP1* gene in the recessive genetic model for *GSTP1* remained non-significant in the adjusted model. The details of the unadjusted and adjusted geometric mean blood Hg concentrations comparison between children with different *GSTP1* genotypes by ASD status are shown in Table 4. We did not find any significant associations with blood Hg concentrations in children with or without ASD in similar unadjusted and adjusted analyses for *GSTM1* and *GSTT1*. Details regarding the *GSTT1* and *GSTM1* results are shown in Table 4.

Further analysis of the significant interactive association of the *GSTP1* gene and ASD status in relation to blood Hg concentrations revealed that while in children with the Val/Val genotype in the co-dominant genetic model there was no significant association between ASD status and blood Hg concentrations (*P* = 0.16), in children with the Ile/Ile and Ile/Val genotypes, there was a significant association between ASD status and blood Hg concentrations (*P* = 0.02 and *P* < 0.01, respectively) after adjusting for the interaction between *GSTP1* and *GSTT1* genes and other aforementioned covariates. We observed similar findings when we used the dominant genetic model for *GSTP1*. Specifically, in the dominant model, while children with ASD who had the Ile/Ile genotype had significantly higher adjusted geometric mean blood Hg concentrations compared to TD controls with the same genotype (0.74 µg/L vs. 0.51 µg/L, *P* = 0.04), ASD cases with either the Ile/Val or Val/Val genotype had an adjusted geometric mean blood Hg concentration of 0.54 µg/L, significantly lower (*P* = 0.01) than that observed for TD control children with the same genotype (0.67 µg/L). Using the recessive genetic model for *GSTP1*, we did not find a significant difference in the adjusted geometric mean blood Hg concentration between ASD cases and TD controls by *GSTP1* genotype. The details of the unadjusted and adjusted geometric mean blood Hg concentrations comparison between ASD cases and TD controls by *GSTP1* genotype are shown in Table 5.

In similar analyses for *GSTT1*, we did not find any significant interactions between ASD status and the *GSTT1* gene in relation to blood Hg concentrations (overall interaction *P* = 0.92 and 0.64 in unadjusted and adjusted models, respectively). Although in the unadjusted model we found ASD cases with the *GSTT1* I/I or I/D genotype had a significantly lower geometric mean blood Hg concentration than TD controls with the same genotypes (0.64 µg/L vs. 0.77 µg/L, *P* = 0.04), this association became non-significant (*P* = 0.70) after adjusting for the interaction between *GSTP1* and *GSTT1* and other aforementioned covariates. Similarly, although the interaction between ASD status and the *GSTM1* gene in relation to blood Hg concentrations was not significant either in the unadjusted (overall interaction *P* = 0.08) or adjusted model (overall interaction *P* = 0.19), in the unadjusted model we found that ASD cases with the *GSTM1* DD genotype had a significantly lower geometric mean blood Hg concentration than TD controls with the same genotypes (0.53 µg/L vs. 0.87 µg/L, *P* <0.01). However, these associations were not significant (*P* = 0.10) after adjusting for the aforementioned covariates. The comparison of the unadjusted and adjusted geometric blood Hg concentrations between ASD cases and TD controls by *GSTM1* and *GSTT1* genotypes are shown in Table 5.

## 4. Discussion

In this study, we have investigated the interactive associations of three metabolic GST genes (*GSTP1*, *GSTT1, and GSTM1*) and ASD status in relation to blood Hg concentrations in Jamaican children, and reported a significant interaction between the *GSTP1* gene and ASD status in relation to blood Hg concentrations using either a co-dominant (overall interaction *P* = 0.002) or dominant (overall interaction *P* = 0.021) genetic model. The interaction between the *GSTP1* gene and ASD status in relation to blood Hg concentrations remained consistently significant in multivariable adjusted analysis after adjusting for the interaction between *GSTP1* and *GSTT1*, child’s age, SES, consumption of leafy vegetables (callaloo, broccoli, or pak choi), fried plantain, and canned fish (sardine or mackerel fish) using either a co-dominant (overall interaction *P* < 0.001) or dominant (*P* = 0.007) genetic model for *GSTP1*. Specifically, after adjusting for the gene-gene interaction between *GSTP1* and *GSTT1*, as well as other aforementioned covariates, ASD cases with genotype Ile/Ile had significantly higher geometric mean blood Hg concentrations than those with genotype Ile/Val (0.73 µg/L vs. 0.48 µg/L, *P* = 0.01) in the co-dominant model, and significantly (though marginally) higher geometric mean blood Hg concentrations than those with the Ile/Val or Val/Val genotypes (0.74 µg/L vs. 0.54 µg/L, *P* = 0.06) in the dominant genetic model. However, in the TD control group, while the geometric mean blood Hg concentration was significantly higher among those with the Ile/Val genotype than those with the Ile/Ile genotype (0.72 µg/L vs. 0.49 µg/L, *P* = 0.03), as well as those with genotype Val/Val (0.72 µg/L vs. 0.51 µg/L, *P* = 0.04) in the co-dominant model, there were no significant associations between genotypes of *GSTP1* and blood Hg concentrations in the dominant model (*P* = 0.11). In addition, after adjusting for the gene-gene interaction between *GSTP1* and *GSTT1* and other aforementioned covariates in the co-dominant model, our findings suggested that while blood Hg concentrations were not significantly different between ASD and TD children who had the Val/Val genotype, the association between ASD status and blood Hg concentrations was significant in children with the Ile/Ile and Ile/Val genotypes. Specifically, among children with the Ile/Ile genotype, ASD cases had significantly higher blood Hg concentrations than TD controls (0.73 µg/L vs. 0.49 µg/L, *P* = 0.02), whereas ASD cases with the Ile/Val genotype had significantly lower blood Hg concentrations than TD controls with the same genotype (0.48 µg/L vs. 0.72 µg/L, *P* < 0.01). Similarly, in the dominant model, among children with the Ile/Ile genotype, ASD cases had a geometric mean blood Hg concentration of 0.74 µg/L that was significantly higher than 0.51 µg/L for TD control children, whereas among children with either an Ile/Val or Val/Val genotype ASD cases had a significantly lower geometric mean blood Hg concentration than TD controls (0.54 µg/L vs. 0.67 µg/L, *P* = 0.01). To our knowledge, we are the first to report an interactive association of *GSTP1* Ile105Val and ASD status in relation to blood Hg concentrations in Jamaican children.

There is evidence suggesting that detoxification of both inorganic and organic Hg in humans depends on their conjugation with glutathione (GSH), a mechanism that relies on GST enzymes [46,79] and polymorphisms in glutathione-related genes [52,53,54,55,80,81,82]. For example, a study in Austria enrolled 324 medical students to investigate associations between mercury exposure and glutathione-related genes including GST gene polymorphisms. Their findings suggested that the *GSTP1* Ala114Val polymorphism was significantly related to mercury body burdens. In addition, they reported synergistic effects of *GSTP1* Ile105Val /glutamate cysteine ligase catalytic subunit (GCLC) and *GSTP1* Ala114Val /*GSTT1* combinations on hair Hg levels compared to single GST gene variants [80]. Another study of 515 dental professionals who were occupationally exposed to Hg reported significant associations between *GSTP1* Ile105Val and Ala114Val polymorphisms and hair Hg levels, as well as between *GSTT1* deletion and urine Hg levels following exposures to elemental mercury via dental amalgams and methylmercury through fish consumption [54]. Similarly, a study that enrolled 905 dental professionals to assess the possible role of single nucleotide polymorphisms (SNPs) in genes that are involved in Hg metabolism in determining Hg concentrations in blood, hair, and urine samples reported a lower blood Hg concentration among variant genotypes for *GSTP1* rs1695 [81]. In our study, although we did not find a significant association between metabolic GST genes in relation to blood Hg concentrations in the additive models, we observed that the role of metabolic GST genes in blood Hg concentrations depends on the ASD status. These findings are consistent with our previous reports of similar significant interactive effects between ASD status and *GSTP1* in relation to blood concentrations of other toxic metals including arsenic [65], and aluminum [66]. All of these findings suggest that genetic variation in metabolic GST genes plays a critical role in individual susceptibility to exposure to neurotoxic metals including Hg because of their consequent variation in detoxification capacity. In addition, our findings support the previous evidence suggesting an impaired detoxification mechanism measured by glutathione-related enzymatic activities in children with ASD compared to TD controls [18,83,84]. Specifically, an age- and sex-matched case-control study in Saudi Arabia reported that compared to TD controls, children with ASD had significantly less active GST enzymes in their blood as determined by the GST-catalyzed reaction between reduced GSH and the GST substrate. They also reported that levels of other markers of detoxification mechanisms including the GSH/ Glutathione disulfide (GSSG) ratio in blood samples of children with ASD were significantly lower than in blood samples of TD controls [84]. Similarly, another study reported lower enzymatic activities of GSH peroxidase in the plasma of children with ASD when compared to TD controls [83]. To the best of our knowledge, there is only one study that investigated the role of GST enzyme activity as the main endogenous detoxifier and cell protector against oxidative stress, along with blood concentrations of toxic heavy metals including Hg in relation to ASD. This case-control study of 52 children with ASD (3-12 years old) and 30 TD controls in Saudi Arabia measured the Hg concentrations in red blood cells, as well as GST activity and vitamin E as enzymatic and non-enzymatic antioxidants in plasma and reported that children with ASD had significantly higher Hg concentrations and lower GST activity and vitamin E concentrations than TD controls. Their findings suggest that accumulation of toxic metals could be a consequence of impaired detoxification in children with ASD [18]. Although our results support the findings of the aforementioned studies about the role of impaired activity of GST enzymes and detoxification capacity in susceptibility of children with ASD to exposure to heavy metals including Hg, genetic information was not available in any of these studies to determine the genotypes associated with less active GST enzymes in children with ASD. Further investigation in other populations is warranted to support our findings suggesting the interactive role of *GSTP1* Ile105Val and ASD status in relation to blood Hg concentrations.

Although in our study we have not measured GSH or GST enzyme activity, the available literature suggests that the mechanism behind the relationship between GST genes and Hg concentrations could be influenced by other factors. As mentioned earlier, both GSH and GST enzymes have a critical role in detoxification of Hg [18,46,79,85]. There is evidence that exposure to Hg depletes GSH, which may impact the balance between oxidants and antioxidants in the central nervous system [86]. Therefore, depletion of GSH and the resulting increase in reactive oxygen species (ROS) is considered as one of the mechanisms responsible for Hg-induced neurotoxicity [87]. Since activity of GST enzymes depends on a steady supply of GSH, in addition to polymorphisms in GST genes, lower GSH levels in children with ASD [83] may also contribute to the decreased activity of GSTs and therefore, make these children more susceptible to the neurotoxic effects of Hg. On the other hand, there is evidence from in vitro studies that in addition to the impact of *GSTP1* genotype on enzyme kinetics, exposure to heavy metals including both inorganic and organic Hg may inhibit *GSTP1* activity in a genotype-dependent manner [88]. These complex relationships may require further investigation.

In our previous study, we reported that the geometric mean blood Hg concentrations of Jamaican children (either in the ASD case or TD control groups) who ate sardine, salt water fish, or mackerel fish, were significantly higher than in those who did not eat these types of fish (all *P* < 0.05) and that these concentrations are about 3.5 times that of children living in the US or Canada [32]. This finding led to changing the policies of Jamaica’s Ministries of Health and Education and the Planning Institute of Jamaica such that Jamaican public schools no longer serve canned fish (sardine or mackerel fish) to children. Since Jamaica is an island nation, fish is an important food in Jamaicans’ traditional diet. In addition, the fishing industry contributes to social and economic development, as well as food security in Jamaica [89]. The average fish consumption per capita is 27.1 kg/year in Jamaica [90] that is about 1.5 times higher than the world’s per capita fish food supply [91]. A study in Jamaica has reported that maternal fish intake was the most significant factor associated with Hg levels in the placenta [92]. Therefore, continuous exposure to Hg is very likely in the Jamaican community via fish consumption. On the other hand, dietary intake of seafood and fish is the main source of omega-3 polyunsaturated fatty acids (PUFA) in humans that play an important role in human health [93], brain development [94] and cognitive performance [95,96,97]. Since our study design is matched case-control and the measurement of exposure to the six metals including mercury took place at the time when the children were evaluated to confirm their ASD status at age 2–8 years, we are unable to establish temporality of the associations we reported in this study. Furthermore, from the currently available data from our ERAJ study it is difficult to establish whether the associations reported could be explained by a causal relationship. For example, although we know that blood Hg concentrations in Jamaican children are much higher than those of children in the US and Canada as a result of consuming larger amounts of fish and seafood [32], in our study we observed in the univariable analysis that children with ASD have lower concentrations of blood Hg than TD children. This finding could be associated with a lower consumption of fish and seafood by children with ASD compared to that of TD children, possibly due to high prevalence of GI issues [98] and food selectivity [99] in children with ASD. On the other hand, lower consumption of fish [100] and seafood [101] could lead to lower levels of omega-3 or higher omega-6 fatty acids in children with ASD that are shown to be important for brain development [94]. In addition, our findings suggested that metabolic GST genes interact with ASD status in relation to blood Hg concentrations and children with specific genetic variants may be more susceptible to Hg exposures due to variations in detoxification capacity. Although, based on our findings we cannot make specific recommendations regarding safe levels of fish consumption in Jamaican children, considering that fish consumption is the main source of omega-3 fatty acids in vulnerable groups (e.g., pregnant women and children), we believe these groups should consume types of fish and seafood with lower risk of Hg contamination. To the best of our knowledge, there is only one recent study in Jamaica that used risk-benefit analysis methods to determine the best fish species for consumption. They measured total mercury, arsenic, selenium, and omega-3 fatty acids (EPA and DHA) in composite samples of 14 fish species that were collected from major fishing villages in Jamaica. Their findings from four risk-benefit analysis methods consistently suggested snappers, parrotfish, doctorfish and cod fish as the best fish species that balance the benefits and risks of seafood consumption in Jamaica based on the levels of Hg, selenium, arsenic, and omega-3 fatty acids [89]. Using findings from the aforementioned study [89], and by conducting additional risk–benefit analysis for seafood and fish consumption in the Jamaican population, we believe that the Jamaica population would benefit from developing evidence-based guidelines and optimal strategies that maximize the benefits of seafood and fish consumption while minimizing the potential risk to this population.

## 5. Limitations

There are several limitations to this study. First, the blood Hg concentration data we had do not differentiate organic and inorganic types of Hg exposure, therefore, detailed discussion about distinct sources of Hg exposure is not provided in this study. However, considering the importance of fish in the traditional diet of Jamaicans, we know that fish consumption is the primary source of Hg exposure in Jamaica. Second, although we measured the frequency of seafood consumption using a standard and culturally appropriate food frequency questionnaire in Jamaica, the questionnaire does not distinguish frequency of consumption of all types of fish separately, which limits assessment of individual associations of each type of fish or seafood with blood Hg concentration in children. For example, we have data on number of “sardine or mackerel fish” servings that children ate per week, but did not assess frequency of eating sardine and mackerel, separately. In addition, we did not have data on prenatal sources of Hg exposure including maternal diet during pregnancy, as well as the levels of other nutrients associated with fish consumption such as PUFA and selenium that play an important role in neurodevelopment and may have a role in the relationship between Hg and ASD. Furthermore, since more TD control children in the ERAJ studies were selected from the Kingston area, they may not represent a random sample of all children in Jamaica, which limits the generalizability of our findings regarding the blood Hg concentrations to children all over Jamaica. We also acknowledge that the observed association between the *GSTP1* rs1695 genotypes and the blood Hg concentration does not necessarily imply that rs1695 is the true causal polymorphism, but may instead be attributable to the effect of another genetic variant that is in linkage disequilibrium with rs1695, though, this was not measured in this study. Moreover, we acknowledge that we have not made any adjustment for multiple comparisons. Therefore, our findings should be replicated in other populations. Furthermore, since the study design is a 1:1 age- and sex-matched case-control, we acknowledge that the associations reported here may not represent causal associations and we advise caution in interpretation of these findings.

## 6. Conclusions

In this paper, we have reported that the ASD cases in Jamaica with *GSTP1* Ile105Val genotype Ile/Ile had significantly higher blood Hg concentrations than those with genotype Ile/Val after accounting for the interaction between *GSTT1* and *GSTP1*, SES, consumption of leafy vegetables, fried plantain, and canned fish (sardine or mackerel fish). However, the geometric mean blood Hg concentration in TD control children with the Ile/Val genotype was significantly higher than those with the Ile/Ile genotype, as well as those with the Val/Val genotype. These findings suggest an interactive association between *GSTP1* and ASD status in relation to blood Hg concentrations that is consistent with the possible role of *GSTP1* in detoxification of Hg. Our finding related to the association of blood Hg concentrations with ASD requires a careful interpretation. Specifically, the findings reported in this study do not imply that Hg exposure is the cause of ASD, but subsets of children with ASD and genotypes that are associated with a higher blood concentration of Hg may potentially be susceptible to further neurodevelopmental impairment based on the available literature. Therefore, our data suggest that the development of targeted interventions focused on dietary and environmental factors could help to moderate exposure to Hg in Jamaican children, especially among those who are more susceptible to adverse outcomes of Hg exposures due to their genetic variants. However, replication of our findings in other populations is warranted.

## Figures and Tables

**Table 1:** Children and Parents’ characteristics by ASD case status (266 matched pairs).

Variables	Categories	ASD Case (*n* = 266)	TD Control (*n* = 266)	*P*-Value *
Child’s sex	Male	217 (81.6)	217 (81.6)	1.00
Child’s age (months)	Age < 72	185 (69.5)	190 (71.4)	0.18
	Age ≥ 72	81 (30.5)	76 (28.6)	
Child’s race	Afro-Caribbean	254 (95.5)	258 (97.0)	0.37
Maternal age ^a^ (at child’s birth)	Age < 35	215 (81.1)	229 (87.7)	0.03
	Age ≥ 35	50 (18.9)	32 (12.3)	
Parental education ^b^ (at child’s birth)	Both up to high school ^†^	98 (37.7)	132 (52.0)	<0.01
	At least one beyond high school ^††^	162 (62.3)	122 (48.0)	
Socioeconomic status (SES)	Car ownership	150 (56.4)	106 (39.9)	<0.01
*GSTP1* ^c^	Ile/Ile	68 (25.9)	65 (24.4)	0.58
	Ile/Val	144 (54.8)	139 (52.3)	
	Val/Val	51 (19.4)	62 (23.3)	
*GSTM1* ^d^	DD ^f^	78 (29.7)	62 (23.6)	0.11
	I/I or I/D ^g^	185 (70.3)	201 (76.4)	
*GSTT1* ^e^	DD ^f^	70 (26.6)	65 (24.8)	0.70
	I/I or I/D ^g^	193 (73.4)	197 (75.2)	
Blood Hg concentration (µg/L) mean (SD) ^h^		1.0 (1.3)	1.0 (0.9)	0.93 **

Data are reported as numbers (percentages), otherwise as indicated. * *P-*Values are based on Wald’s test in conditional logistic regression models. ** *P*-value is based on Related-Samples Wilcoxon Signed Rank Test that compares the distribution of blood aluminum concentration between ASD case and TD control groups; ^†^ Up to high school education means attended Primary/Jr. Secondary, and Secondary/High/Technical schools. ^††^ Beyond high school education means attended a Vocational, Tertiary College, or University. ^a^ Maternal age was missing for 1 ASD case and 5 TD controls. ^b^ Parental education was missing for 6 ASD cases and 12 TD controls. ^c^ *GSTP1* genotype was missing for 3 ASD cases. ^d^ *GSTM1* genotype was missing for 3 ASD cases and 3 TD controls. ^e^ *GSTT1* was missing for 3 ASD cases and 4 TD controls. ^f^ DD indicates the null alleles for *GSTT1* and *GSTM1*. ^g^ I/I or I/D indicate the homozygote (I/I) or a heterozygote (I/D) for *GSTT1* and *GSTM1*. ^h^ Hg: Mercury arithmetic mean.

**Table 2:** Associations between dietary consumption and ASD case status using Conditional Logistic Regression (CLR) (266 matched pairs).

Exposure Variables	Category		ASD Case *n* (%)	TD Control *n* (%)	MOR	95% CI	*P*-Value ^c^
Source of drinking water ^a^	Piped water		207 (77.8)	226 (85.3)	0.78	(0.39, 1.56)	0.48
Source of water for cooking ^a^	Piped water		244 (91.7)	252 (95.1)	0.55	(0.26, 1.15)	0.11
Fruits and vegetables consumption ^b^	Root vegetables	Yam, sweet potato, or dasheen	154 (58.1)	174 (65.4)	0.71	(0.49, 1.02)	0.07
		Carrot or pumpkin	204 (77.0)	231 (86.8)	0.52	(0.33, 0.82)	0.01
	Leafy vegetables	Lettuce	119 (44.9)	167 (62.8)	0.42	(0.28, 0.63)	<0.01
		Callaloo, broccoli, or pakchoi	192 (72.4)	223 (83.8)	0.48	(0.31, 0.76)	<0.01
		Cabbage	128 (48.3)	157 (59.0)	0.60	(0.42, 0.88)	<0.01
	Fruits	Tomatoes	151 (56.9)	196 (73.7)	0.46	(0.32, 0.68)	<0.01
		Ackee	119 (44.9)	182 (68.4)	0.32	(0.21, 0.48)	<0.01
		Avocado	110 (41.5)	173 (65.0)	0.34	(0.23, 0.51)	<0.01
		Green banana	153 (57.7)	180 (67.7)	0.63	(0.44, 0.91)	0.01
		Fried plantains	189 (71.3)	228 (85.7)	0.42	(0.27, 0.66)	<0.01
Seafood consumption	High seafood consumption (more than 6 meals per week)		58 (21.8)	76 (28.6)	0.67	(0.44, 1.02)	0.06
	Ate salt water fish		170 (63.9)	185 (69.6)	0.73	(0.49, 1.10)	0.13
	Ate fresh water fish (pond fish, tilapia)		95 (35.7)	86 (32.3)	1.20	(0.81, 1.77)	0.37
	Ate sardine, mackerel (canned fish)		200 (75.2)	227 (85.3)	0.53	(0.34, 0.82)	<0.01
	Ate tuna (canned fish)		80 (30.1)	92 (34.6)	0.80	(0.55, 1.17)	0.25
	Ate salted fish (pickled mackerel)		175 (65.8)	214 (80.5)	0.49	(0.33, 0.72)	<0.01
	Ate shellfish (lobsters, crabs)		14 (5.3)	36 (13.5)	0.35	(0.18, 0.68)	<0.01
	Ate shrimp		30 (11.3)	46 (17.3)	0.63	(0.39, 1.02)	0.06

**^a^** Data missing for one TD control; **^b^** For all variables under fruits and vegetables consumption data were missing for one ASD case; ^c^ *P*-values are based on Wald’s test in conditional logistic regression models that compares the distribution of dietary consumption between ASD case and TD control groups.

**Table 3:** Associations of various independent variables with blood mercury concentrations based on univariable General Linear Models (266 matched pairs).

Variables	Category		Yes		No		*P*-Value **
			Mean Hg * (μg/L)	*N*	Mean Hg * (μg/L)	*N*	
ASD status	Autism Spectrum Disorder		0.62	266	0.76	266	<0.01
Child’s age (months)	Age ≥ 72		1.07	157	0.57	375	0.05
Child’s sex	Male		0.71	434	0.62	98	0.16
Socioeconomic status (SES)	Own a car		0.64	256	0.73	276	0.20
Maternal age ^a^ (at child’s birth)	≥35 years		0.55	82	0.71	444	0.06
Parental education levels ^b^ (at child’s birth)	At least one of the parents had education beyond high school		0.63	284	0.75	230	0.06
Source of drinking water ^c^	Piped water		0.69	495	0.65	36	0.79
Fruits and vegetables consumption ^d^	Root vegetables	Yam, sweet potato, or dasheen	0.75	328	0.60	203	0.03
		Carrot or pumpkin	0.70	435	0.65	96	0.60
	Leafy vegetables	Lettuce	0.75	286	0.62	245	0.10
		Callaloo, broccoli, or pak choi	0.74	415	0.55	116	0.02
		Cabbage	0.77	285	0.61	246	0.03
	Fruits	Tomatoes	0.77	347	0.56	184	<0.01
		Ackee	0.75	301	0.62	230	0.08
		Avocado	0.76	283	0.62	248	0.06
		Green banana	0.74	333	0.61	198	0.06
		Fried plantains	0.74	417	0.53	114	<0.01
Seafood consumption	High seafood consumption (more than 6 meals per week)		0.87	134	0.63	398	<0.01
	Ate salt water fish		0.79	355	0.52	177	<0.01
	Ate fresh water fish (pond fish, tilapia)		0.76	181	0.65	351	0.19
	Ate sardine, mackerel (canned fish)		0.74	427	0.50	105	<0.01
	Ate tuna (canned fish)		0.79	172	0.64	360	0.07
	Ate salted fish (pickled mackerel)		0.73	389	0.58	143	0.03
	Ate shellfish (lobsters, crabs)		0.87	50	0.67	482	0.14
	Ate shrimp		0.80	76	0.67	456	0.19
Genes ^e^	*GSTT1* (I *) ^f^		0.70	384	0.66	134	0.61
	*GSTM1* (I *) ^f^		0.70	382	0.67	138	0.71
	*GSTP1* (Ile/Ile) ^g^		0.77	132	0.66	394	0.17
	*GSTP1* (Val/Val) ^g^		0.59	111	0.72	415	0.14
	*GSTP1* (Ile/Val) ^g^		0.69	283	0.69	243	0.97

* Mean Hg indicates the geometric mean of mercury = Exp. [Mean (ln Hg)]; ** *P*-values are based on GLMs that compare geometric mean blood mercury concentrations between children who had the characteristic described (in the “Yes” column) and those who did not (in the “No” column); The “Yes” column includes participants who had the characteristic described for the categories in each variable; The “No” column includes participants who did not have the characteristic described for the categories in each variable; ^a^ Maternal age was missing for 6 participants; ^b^ Parental education level was missing for 18 participants; ^c^ Source of drinking water was missing for one participant; ^d^ Fruits and vegetables consumption was missing for one participant; ^e^ Results based on 259 matched pairs for *GSTT1*, 260 pairs for *GSTM1*, and 263 pair for *GSTP1*; ^f^ I* indicates the homozygote (I/I) or a heterozygote (I/D) for *GSTT1* and *GSTM1*; ^g^ *GSTP1* has three categories (Ile/Ile, Ile/Val, and Val/Val).

**Table 4:** Unadjusted and adjusted geometric mean blood mercury concentrations by GST genotypes based on General Linear Models (GLM) that include interaction between GST genes and ASD case status (ASD and TD control) *.

Models	Gene	(Column A) Genotypes Compared	Referent Genotypes	Group	Unadjusted (μg/L) ^a^			Adjusted (μg/L) ^b^		
					Geometric Mean Hg of Children with Genotypes in Column A ^c^	Geometric Mean Hg of Children with Referent Genotypes ^c^	*P*-Value ^d^	Geometric Mean Hg of Children with Genotypes in Column A ^c^	Geometric Mean Hg of Children with Referent Genotypes ^c^	*P*-Value ^d^
Co-dominant ^e†^	*GSTP1*	Ile/Ile	Ile/Val	TD Control	0.70	0.89	0.16	0.49	0.72	0.03
	*GSTP1*	Ile/Ile	Ile/Val	ASD Case	0.87	0.54	<0.01	0.73	0.48	0.01
	*GSTP1*	Ile/Ile	Val/Val	TD Control	0.70	0.57	0.28	0.49	0.51	0.85
	*GSTP1*	Ile/Ile	Val/Val	ASD Case	0.87	0.62	0.11	0.73	0.66	0.62
	*GSTP1*	Ile/Val	Val/Val	TD Control	0.89	0.57	<0.01	0.72	0.51	0.04
	*GSTP1*	Ile/Val	Val/Val	ASD Case	0.54	0.62	0.45	0.48	0.66	0.10
Dominant ^f⸶^	*GSTP1*DOM	Ile/Val or Val/Val	Ile/Ile	TD Control	0.78	0.70	0.53	0.67	0.51	0.11
	*GSTP1*DOM	Ile/Val or Val/Val	Ile/Ile	ASD Case	0.56	0.85	0.01	0.54	0.74	0.06
Recessive ^g⸷^	*GSTP1*REC	Val/Val	Ile/Ile or Ile/Val	TD Control	0.57	0.83	0.03	0.51	0.64	0.20
	*GSTP1*REC	Val/Val	Ile/Ile or Ile/Val	ASD Case	0.63	0.63	0.99	0.66	0.56	0.37
Recessive ^⸸^	*GSTT1*	I/I or I/D	DD	TD Control	0.77	0.74	0.79	0.61	0.63	0.83
		I/I or I/D	DD	ASD Case	0.64	0.59	0.67	0.59	0.55	0.67
Recessive ^‡^	*GSTM1*	I/I or I/D	DD	TD Control	0.73	0.87	0.30	0.63	0.66	0.81
		I/I or I/D	DD	ASD Case	0.66	0.53	0.14	0.63	0.49	0.09

* Results based on 263 pair for *GSTP1*, 260 pairs for *GSTM1*, and 259 matched pairs for *GSTT1*. ^a^ In the unadjusted GLMs, the independent variables include pairs, ASD status, GST gene, and GST gene interaction with ASD; ^b^ In multivariable GLMs in addition to the variables in the unadjusted model we adjusted for Child’s age, socioeconomic status, consumption of callaloo, broccoli, or pak choi, fried plantain, and sardine or mackerel fish. Additionally, we account for the interaction between *GSTT1* and *GSTP1* in relation to blood Hg concentrations in adjusted models related to *GSTP1* and *GSTT1* genes; ^c^ Mean Hg indicates the geometric mean of mercury = Exp. [Mean (ln Hg)]; ^d^ *P*-values are for the comparison of mean blood mercury concentrations of children with genotypes in “Column A” compared to those with “referent genotypes”, stratified by ASD case status (ASD and TD control), based on CONTRAST option in the SAS program for GLMs as described in the Methods section; ^e^ *GSTP1* in the co-dominant model has three categories (Ile/Ile, Ile/Val, and Val/Val); ^f^ *GSTP1* (DOM) = *GSTP1* in the dominant model has two categories (Val/Val or Ile/Val, Ile/Ile); ^g^ *GSTP1* (REC) = *GSTP1* in the recessive model has two categories (Val/Val, Ile/Ile or Ile/Val). ^†^ Overall interaction *P* = 0.002 and *P* < 0.001 for unadjusted and adjusted models, respectively. ⸶ Overall interaction *P* = 0.021 and *P* = 0.007 for unadjusted and adjusted models, respectively. ⸷ Overall interaction *P* = 0.112 and *P* = 0.081 for unadjusted and adjusted models, respectively. ⸸ Overall interaction *P* = 0.916 and *P* = 0.645 for unadjusted and adjusted models, respectively. ^‡^ Overall interaction *P* = 0.080 and *P* = 0.193 for unadjusted and adjusted models, respectively.

**Table 5:** Unadjusted and adjusted geometric mean blood mercury concentrations by ASD status (ASD and TD control) based on General Linear Models (GLM) that includes interaction between GST genotypes and ASD case status (ASD and TD control) *.

Gene	Models	(Column A) Group Compared	Referent Group	*GSTP1* Genotypes	Unadjusted Model (μg/L) ^a^			Adjusted Model (μg/L) ^b^		
					Geometric Mean Hg of Children with Group Compared in Column A ^c^	Geometric Mean Hg of Children with Referent Group ^c^	*P*-Value ^d^	Geometric Mean Hg of Children with Group Compared in Column A ^c^	Geometric Mean Hg of Children with Referent Group ^c^	*P*-Value ^d^
*GSTP1*	Co-dominant ^e†^	ASD Case	TD Control	Ile/Ile	0.87	0.70	0.24	0.73	0.49	0.02
		ASD Case	TD Control	Ile/Val	0.54	0.89	<0.01	0.48	0.72	<0.01
		ASD Case	TD Control	Val/Val	0.62	0.57	0.63	0.66	0.51	0.16
	Dominant ^f⸶^	ASD Case	TD Control	Ile/Ile	0.85	0.70	0.29	0.74	0.51	0.04
		ASD Case	TD Control	Val/Val or Ile/Val	0.56	0.78	<0.01	0.54	0.67	0.01
	Recessive ^g⸷^	ASD Case	TD Control	Val/Val	0.63	0.57	0.65	0.66	0.51	0.10
		ASD Case	TD Control	Ile/Ile or Ile/Val	0.63	0.83	<0.01	0.56	0.64	0.21
*GSTT1*	Recessive^⸸^	ASD Case	TD Control	DD	0.59	0.74	0.24	0.55	0.63	0.44
		ASD Case	TD Control	I/I or I/D	0.64	0.77	0.04	0.59	0.61	0.70
*GSTM1*	Recessive^‡^	ASD Case	TD Control	DD	0.53	0.87	<0.01	0.49	0.66	0.10
		ASD Case	TD Control	I/I or I/D	0.66	0.73	0.29	0.63	0.63	0.94

* Results based on 263 pair for *GSTP1*, 260 pairs for *GSTM1*, and 259 matched pairs for GSTT1. ^a^ In the unadjusted GLMs, the independent variables include pairs, ASD status, GST gene, and GST gene interaction with ASD; ^b^ In multivariable GLMs in addition to the variables in the unadjusted model we adjusted for Child’s age, socioeconomic status, consumption of callaloo, broccoli, or pak choi, fried plantain, and sardine or mackerel fish. Additionally, we account for the interaction between *GSTT1* and *GSTP1* in relation to blood Hg concentrations in adjusted models related to *GSTP1* and *GSTT1* genes; ^c^ Mean Hg indicates the geometric mean of mercury = Exp. [Mean (ln Hg)]; ^d^ *P*-values are for the comparison of mean blood mercury concentrations of children with the ASD case status in “Column A” compared to those with the TD control status in “referent group”, stratified by GST genotypes, based on CONTRAST option in the SAS program for GLMs as described in the Methods section; ^e^ *GSTP1* in the co-dominant model has three categories (Ile/Ile, Ile/Val, and Val/Val); ^f^ *GSTP1* (DOM) = *GSTP1* in the dominant model has two categories (Val/Val or Ile/Val, Ile/Ile); ^g^ *GSTP1* (REC) = *GSTP1* in the recessive model has two categories (Val/Val, Ile/Ile or Ile/Val). ^†^ Overall interaction *P* = 0.002 and *P* < 0.001 for unadjusted and adjusted models, respectively. ⸶ Overall interaction *P* = 0.021 and *P* = 0.007 for unadjusted and adjusted models, respectively. ⸷ Overall interaction *P* = 0.112 and *P* = 0.081 for unadjusted and adjusted models, respectively. ⸸ Overall interaction *P* = 0.916 and *P* = 0.645 for unadjusted and adjusted models, respectively. ^‡^ Overall interaction *P* = 0.080 and *P* = 0.193 for unadjusted and adjusted models, respectively.

## Data Availability

The data analyzed in this study are from two grants (i.e., R21 and R01). The data from R01 is or will be publicly available through National Database for Autism Research (NDAR) via the following link: https://nda.nih.gov/edit_collection.html?id=2063. Data from R21 will also be available upon request from the corresponding author based on the following data sharing agreement stated in the R21 grant: (1) a commitment to using the data only for research purposes and not to identify any individual participant; (2) a commitment to using best statistical and ethical practices in analyzing and reporting finding; (3) a commitment to securing the data using appropriate information technology; (4) a commitment to crediting the source and the funding agencies of the original project in all publications and presentations; and (5) a commitment to destroying or returning the data after analyses are completed.
